# Health professionals for global health: include dental personnel upfront!

**DOI:** 10.3402/gha.v6i0.21398

**Published:** 2013-07-16

**Authors:** Raman Preet

**Affiliations:** Epidemiology and Global Health, Department of Public Health and Clinical Medicine, Umeå Centre for Global Health Research, Umeå University, Umeå, Sweden

**Keywords:** health professionals, oral health, inclusion, global health

## Abstract

The Global Health Beyond 2015 was organized in Stockholm in April 2013, which was announced as public engagement and where the dialogue focused on three main themes: social determinants of health, climate change and the non-communicable diseases. This event provided opportunity for both students and health professionals to interact and brainstorm ideas to be formalized into Stockholm Declaration on Global Health. Amongst the active participation of various health professionals, one that was found significantly missing was that of oral health. Keeping this as background in this debate, a case for inclusion of oral health professions is presented by organizing the argument in four areas: education, evidence base, political will and context and what each one offers at a time when Scandinavia is repositioning itself in global health.

When the Swedish Society of Medicine organized an event, Global Health Beyond 2015, in April 2013 in Stockholm with the intended outcome of producing a Stockholm Declaration on Global Health (1), the meeting was profiled as public engagement (2). Not to be confused with yet another high-level meeting on global health (2), this dialogue focused on three main themes: social determinants of health, climate change, and non-communicable diseases (NCDs) (1). There was opportunity for both students and health professionals to interact and brainstorm ideas for action plans related to both educational and research challenges, and to offer those ideas to be incorporated into the Stockholm Declaration. This recently published declaration is intended to serve as an influential framework guiding Scandinavia and other countries to promote equitable global health and development by strengthening capacities and adopting agendas for global healthy living (3).

During the event, there was an energetic exchange among a community representing various disciplines like medicine, nursing, midwifery, public health, engineering, ergonomics, anthropology, and social science. Notably missing were any from the oral health professions. Connecting the mouth and oral health with the rest of the body, as well as with population health, seems to be forgotten in the current dialogue about health for all. Understanding why this is so, and what can be undertaken to correct that disconnect and enhance inclusivity, is the task at hand.

To argue that all health professionals, and for that matter the general public as well, have stakes in the outcome of such a deliberation is to argue for our own health and our own national, regional, and global self-interests. The case for inclusion of one particular major body of health professionals is being offered now by establishing the arguments in four categories – health professions’ education, evidence base, political will, and the context in which Scandinavia addresses its own vision of global health.

## Education

While in much of Europe, stomatologists have been historically a specialty of medicine, modern dentists evolved through a separate pathway in educational environments apart from medical schools. Unfortunately, in these separate pathways, there has been minimal opportunity for each to learn the curricular content of the other, and a lack of integration of the oral cavity with the rest of the body's systems then translates into practices that are separate and distinct territories for professional activity. It is no wonder that when population health is being considered, neither community begins its discussions with the other being equal partners. Yet, precisely in schools of public health and in ministries of health, there are unique encounters where integration can and often does occur – given, of course, appropriate leadership and vision for achieving disease prevention and health promotion for all, measured at a population level. The fact that more and more integration is where global health is heading is supported by initiatives to address interprofessional education models, building upon the Lancet Commission report (4) that is now being explored in universities in several parts of the world, notably in the United States and Canada (5).

## Evidence base

In May 2013, Marcenes et al. reported that oral conditions affected 3.9 billion people and that untreated dental caries in permanent teeth alone were the most prevalent condition assessed among all of the 291 measured global disease entities (6). Dental caries is known to be the most common non-communicable disease of children worldwide (7). Periodontal disease, a common chronic inflammatory disease of the gums, has been associated with cardiovascular disease, diabetes, as well as rheumatoid arthritis. Other relationships with pregnancy, osteoporosis, and cancer abound in the literature (8). Oral cancer is the eighth most common cancer among men in the world, and the use of tobacco and excessive intake of alcohol are two of the major risk factors. Oral bacteria have been shown to increase the risk of respiratory infections (7). These facts alone should push the case forward for oral health professionals to be upfront as they work with other health professionals at tackling the common risk factors for these major diseases (7, 9, 10). All of the diseases, whether infectious or non-communicable, also share the same causes, with societal influences stemming from poverty, poor access to education, unhealthy foods, inadequate transportation, and the like. Oral health inequalities mirror those in general health. It is an established fact that the oral health of lower socioeconomic status (SES) groups is worse than that of their higher SES counterparts. This social gradient is a shared phenomenon with general health (11). Finally, is climate change related to oral health? There is some relationship because climate impacts nutrition and food availability, elements that affect all aspects of health where there is a connection with foods. It is well documented that fluoride, as a trace element and nutrient, impacts dental decay and the integrity of bone tissue. In relation to environmental concerns, United Nations Environment Programme (UNEP) negotiations among 140 countries known as the Minamata Convention, held in January 2013, adopted a treaty on reducing harm from mercury (12). The recommendation to phase down the use of mercury amalgam dental fillings should accompany stepping up efforts to prevent dental caries as well as research to find alternative effective and long-lasting dental restorative materials.

## Political will

The case for putting teeth into the NCDs was effectively summarized at a side event to the first UN Global Summit on NCDs in New York City, September 19, 2011, where essential links of association between and among heart, cancer, diabetes, and respiratory diseases in relationship to dental caries, periodontal diseases, oral cancer, and orofacial trauma were made. The administrator of the UN Development Program, Helen Clark, made these key statements at the same event: ‘Our challenge is translating knowledge about the drivers of poor health into action – within both the development and health communities …. A focus on oral health in overall primary health care will not only help improve oral health itself, but will also reduce the rate of cardiovascular diseases, cancer, chronic respiratory diseases, and diabetes’ (13). This is the first time ever that a high-level political spokesperson articulated the case for integrating oral health into development assistance, and, in the end, 193 member states included in Article 19 of their Political Declaration on the Prevention and Control of NCDs that ‘renal, oral and eye diseases pose a major burden for many countries and that these diseases share common risk factors and can benefit from common responses to non-communicable diseases’ (14).

## Context

Sweden as a nation has much to offer in terms of public health action. Altruism and associated values that contribute to making a small difference in the world are strong in Swedish society. Swedes might have some doubts about its role in global health, but Sweden is a global leader in health, according to Richard Horton in a comment on the Stockholm event in *The Lancet* (2). He mentions individuals like Hans Rosling and Mariam Claeson, who are well known in the field of global health, and also accredits Umeå University as an eminent academic center in global health (2).

Sweden developed its Public Dental Service in 1938, and by the 1960s dental hygienists, as a professional category, were added for delivering free dental care, primarily to children (15). Sweden has a well-established oral public health delivery system, as a result of which Swedes on average enjoy good oral health. Currently, in northern Sweden, there is a community health intervention entitled *Salut*, which is designed to improve child health promotion practices in multiple sectors in which dental health services are embedded as an integral part; it is one example, and findings from it may inform planning for integrated primary care in other platforms (16). When Scandinavia is developing a renewed vision for itself in global health, the opportunity to be inclusive of the oral health professional workforce is not only timely but also critical to successful health outcomes, not just those specific to the oral craniofacial complex but also the whole-body system. The positive impact will clearly be on total health.

## Way forward toward inclusion

Focusing on diseases is akin to focusing on the outcome and not looking at the causes. Unless the causes and the associated risk factors are tackled systematically, the diseases will persist *ad infinitum*. Instead, we could be trying to set targets and indicators on common risk factors, and perhaps even on the causes of the causes (i.e. the social determinants), and move away from vertically set targets and indicators focusing on one disease at a time.

Or, we could reorient the health professions’ education to address the needs of the context in which services would be delivered, and prepare students to become not only competent technical experts but also culturally competent and sensitive to what can be enhanced in a health-promoting environment (4, 17).

Gunhild Stordalen, a climate activist and philanthropist, asks for better integration among academia, enterprise, governments, and international bodies to deal with the public health impact of climate change (18). Indeed, this concept of integration applies to all the fields working for global health. The Stockholm Declaration for Global Health also urges the linking of existing agendas with new ones and for exploiting opportunities and synergies to act for global health (3). On that note, for ‘closing the gap in a generation’ (19), there is a need for creating new understanding that allows better integration of all health expertise to focus away from the downstream consequences and collaborate and coordinate toward upstream causes. Would not a more constructive approach to moving forward in global health be to include oral health professionals as early in the process as possible, and would not that enlarged workforce accelerate positive change?

## References

[1] Byass P, Friberg P, Blomstedt Y, Wall S (2013). Beyond 2015: time to reposition Scandinavia in global health?. Glob Health Action.

[2] Horton R (2013). Offline: the Stockholm syndrome. Lancet.

[3] Friberg P, Wall S, Blomstedt Y, Beaglehole R, Bonita R, Stordalen G (2013). Public and global engagement with global health. Lancet.

[4] Frenk J, Chen L, Bhutta ZA, Cohen J, Crisp N, Evans T (2010). Health professionals for a new century: transforming education to strengthen health systems in an interdependent world. Lancet.

[5] Formicola AJ, Andrieu SC, Buchanan JA, Childs GS, Gibbs M, Inglehart MR (2012). Interprofessional education in U.S. and Canadian dental schools: an ADEA team study group report. J Dent Educ.

[6] Marcenes W, Kassebaum NJ, Bernabé E, Flaxman M, Naghavi A, Lopez A (2013). Global burden of oral conditions in 1990–2010: a systematic analysis. J Dent Res.

[7] Beaglehole R, Benzian H, Crail J, Mackay J (2009). The oral health atlas: mapping a neglected global health issue.

[8] Genco RJ, Willams RC (2010). Periodontal disease and overall health: a clinician's guide.

[9] Cohen LK, Benzian H, Bergman M (2012). UN summit: stepping up efforts to address oral diseases, global health through oral health. Compendium.

[10] Sheiham A, Watt R (2002). The common risk factor approach: a rational approach for promoting health. Community Dent and Oral Epidemiol.

[11] Watt R (2007). From victim blaming to upstream action: tackling the social determinants of oral health inequalities. Community Dent Oral Epidemiol.

[12] Kirby T (2013). World report: UN agrees new treaty to reduce harm from mercury. Lancet.

[13] Statement by Helen Clark UNDP Administrator: UN Summit on non-communicable diseases side event: putting teeth into the NCDs. http://www.undp.org/content/undp/en/home/presscenter/speeches/2011/09/19/statement-by-helen-clark-undp-administrator-un-summit-on-non-communicable-diseases-side-event-putting-teeth-into-the-ncds/.

[14] United Nations General Assembly Political declaration of the high-level meeting of the General Assembly on the prevention and control of non-communicable diseases. Resolution A/66/L1.2011.

[15] Swedish Public Dental Service http://www.folktandvarden.se/in-english/about-the-swedish-public-dental-service.

[16] Edvardsson K, Ivarsson A, Garvare R, Eurenius E, Lindkvist M, Mogren I (2012). Improving child health promotion practices in multiple sectors – outcomes of the Swedish Salut Programme. BMC Public Health.

[17] Seymour B, Muhumuza I, Mumena C, Isyagi M, Barrow J, Meeks V (2013). Including oral health training in a health system, strengthening program in Rwanda. Glob Health Action.

[18] Stordalen GA, Rocklöv J, Nilsson M, Byass P (2013). Only an integrated approach across academia, enterprise, governments and global agencies can tackle the public health impact of climate change. Glob Health Action.

[19] Commission on the Social Determinants of Health (2008) Closing the gap in a generation: health equity through action on the social determinants of health.

